# Long-latency modulation of motor cortex excitability by ipsilateral posterior inferior frontal gyrus and pre-supplementary motor area

**DOI:** 10.1038/srep38396

**Published:** 2016-12-08

**Authors:** Francesca Fiori, Emilio Chiappini, Marco Soriano, Riccardo Paracampo, Vincenzo Romei, Sara Borgomaneri, Alessio Avenanti

**Affiliations:** 1 Dipartimento di Psicologia and Centro studi e ricerche in Neuroscienze Cognitive, Campus di Cesena, Università di Bologna 47521 Cesena, Italy; 2 IRCCS Fondazione Santa Lucia, 00179 Rome, Italy; 3 Dipartimento di Psicologia, Sapienza Università di Roma, 00185 Rome, Italy; 4 Centre for Brain Science, Department of Psychology, University of Essex, UK

## Abstract

The primary motor cortex (M1) is strongly influenced by several frontal regions. Dual-site transcranial magnetic stimulation (dsTMS) has highlighted the timing of early (<40 ms) prefrontal/premotor influences over M1. Here we used dsTMS to investigate, for the first time, longer-latency causal interactions of the posterior inferior frontal gyrus (pIFG) and pre-supplementary motor area (pre-SMA) with M1 at rest. A suprathreshold test stimulus (TS) was applied over M1 producing a motor-evoked potential (MEP) in the relaxed hand. Either a subthreshold or a suprathreshold conditioning stimulus (CS) was administered over ipsilateral pIFG/pre-SMA sites before the TS at different CS-TS inter-stimulus intervals (ISIs: 40–150 ms). Independently of intensity, CS over pIFG and pre-SMA (but not over a control site) inhibited MEPs at an ISI of 40 ms. The CS over pIFG produced a second peak of inhibition at an ISI of 150 ms. Additionally, facilitatory modulations were found at an ISI of 60 ms, with supra- but not subthreshold CS intensities. These findings suggest differential modulatory roles of pIFG and pre-SMA in M1 excitability. In particular, the pIFG –but not the pre-SMA– exerts intensity-dependent modulatory influences over M1 within the explored time window of 40-150 ms, evidencing fine-tuned control of M1 output.

Interactions between premotor and motor brain regions are critical for understanding motor network functioning. The posterior inferior frontal cortex (including the posterior sector of the inferior frontal gyrus, pIFG, and the ventral premotor cortex, vPMc) and the supplementary motor complex (including the pre-supplementary motor area, pre-SMA, and the supplementary motor area, SMA) are key regions within the motor system linking cognition to action^1^,^2^,^3^,^4^,^5^,^6^,^7^,^8^,^9^. Both inferior frontal and supplementary areas have sparse projections to the spinal cord, whereas their most posterior premotor sectors (i.e., vPMc and SMA) possess extensive projections to the primary motor cortex (M1) to influence motor output^10^,^11^,^12^,^13^,^14^. Such projections appear less abundant in the most rostral sectors of inferior frontal and supplementary regions, particularly in the pre-SMA, which appears to exert its influence over motor ouput via indirect interconnected pathways^10^,^14^,^15^. Yet, rostral premotor regions appear critical for motor functions, and neuroimaging and neurophysiological studies indicate strong connectivity between rostral premotor cortices and M1^1^,^2^,^4^,^6^.

Functional imaging studies have highlighted premotor-motor functional coupling at rest^16^,^17^ and disruption of this coupling in a number of neurological conditions affecting the motor system^18^,^19^. However, these functional connectivity studies rely on an approach that is correlational in nature and characterized by low temporal resolution. Therefore, brain stimulation techniques might be better suited for highlighting the time-course of rostral premotor-M1 causal interactions.

Dual-site transcranial magnetic stimulation (dsTMS) is a valuable neurophysiological method for non-invasively mapping causal connectivity with high temporal resolution^20^,^21^,^22^,^23^,^24^. In the dsTMS protocol, a conditioning stimulus (CS) is administered over a target (e.g., premotor) region to activate hypothetical pathways (through direct or indirect connections) from the site of stimulation to M1. The CS is followed by a test stimulus (TS) that is administered over M1 to induce motor-evoked potentials (MEPs) in contralateral muscles. Both facilitation and inhibition may occur at the TS site (i.e., M1), evidencing different neurophysiological interactions between the stimulated areas depending on CS intensity and the interstimulus intervals (ISIs) between CS and TS.

The dsTMS paradigm has been extensively used to investigate interhemispheric connections between homologous M1 sites^22^,^23^,^25^,^26^,^27^. More recently, interactions between non-primary motor areas and M1 have started to be investigated^20^,^28^,^29^,^30^. Using dsTMS, studies have focused on how M1 excitability is influenced by a CS administered over posterior inferior frontal cortices^20^,^31^,^32^,^33^,^34^ and the supplementary motor complex^15^,^20^,^21^,^35^. These studies have focused on short-latency connectivity using various ISIs of <15 ms, and have shown that a CS over premotor areas can modulate MEPs induced by the TS over M1 only at specific ISIs of ~4–8 ms, evidencing time-dependent effects. Moreover, these studies suggest that the excitatory or inhibitory nature of premotor-to-motor short-latency interactions depends on TMS intensity, as partially distinct neural populations are recruited depending on TMS intensities. For example, Bäumer and colleagues^33^ showed that a relatively low subthreshold CS over posterior inferior frontal regions (80% of active motor threshold; aMT) and a higher intensity CS (90% of resting motor threshold; rMT) produced facilitation and inhibition of MEPs, respectively. These findings highlighted the intensity- and time-dependent nature of short-latency premotor-motor interactions.

Previous dsTMS studies have mainly used short ISIs to explore ipsilateral premotor-motor interactions. However, neural interactions within the motor system likely occur on different time-scales. Indeed, longer-latency interactions with ISIs up to 150 ms have been documented between M1 and contralateral motor-related areas^36^,^37^ and studies have shown altered long-latency M1-M1 interhemispheric interactions (at an ISI of 40 ms) in neurological conditions affecting motor control^25^,^26^. Thus, motor network functioning may be based on optimal tuning between short-latency, as well as long-latency, interactions.

The goal of this study was to explore, for the first time, the dynamics of long-latency rostral premotor-motor interactions. To this aim, we used dsTMS over pIFG-M1 and pre-SMA-M1 circuits, and tested the effect of ISI (between 40 and 150 ms) and CS intensity on MEP amplitude modulation (Fig. 1). Our findings show that long-latency functional connections do exist between rostral premotor and motor areas, and that specific time intervals and intensities are crucial for observing causal influences of pIFG and pre-SMA over M1 excitability during a resting state. Although these interactions likely involve indirect pathways, tracking the time-course of long-latency pIFG-M1 and pre-SMA-M1 interactions is important not only for understanding cortico-cortical connectivity (and its disruption in clinical conditions), but also for developing novel information-based^38^ non-invasive transcranial brain stimulation methods aimed at manipulating connectivity, such as the cortico-cortical paired associative stimulation (ccPAS) protocol^38^,^39^,^40^,^41^,^42^ which relies on the critical ISIs identified by dsTMS.

## Results

**Figure 1:**
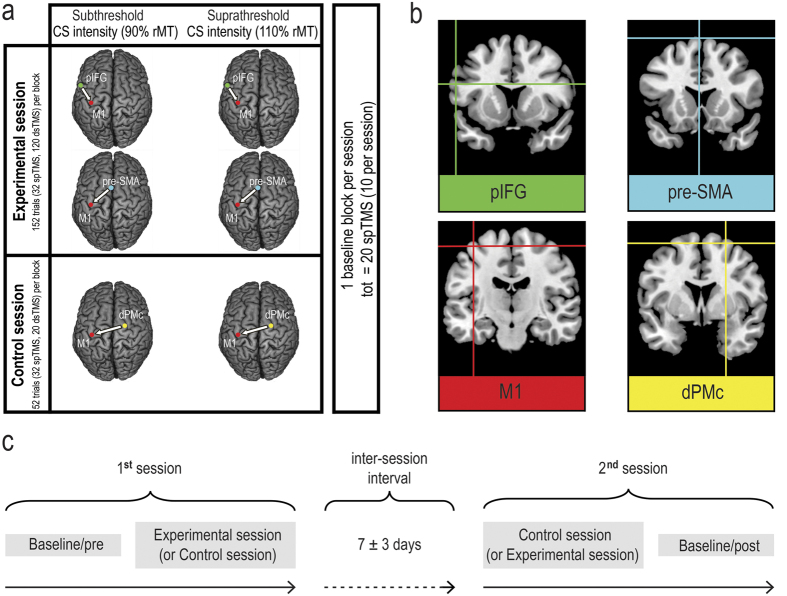
(**a**) Schematic representation of the experimental and control sessions. For each experimental and control block, brain stimulation sites, CS intensity and number of trials are reported. The baseline consisted of a total of 20 spTMS trials, recorded during the experimental session (10 MEPs) and the control session (10 MEPs). The baseline trials were collected at the beginning or at the end of each session. (**b**) Brain stimulation sites. Coordinates in Talairach space corresponding to the projection of the stimulated scalp sites on the brain surface were estimated through neuronavigation software (left mean pIFG coordinates ± S.D.: x = −54.8 ± 1.3, y = 9.1 ± 1.0, z = 24 ± 1.0; pre-SMA: x = 0.1 ± 0.3, y = 9.8 ± 6.5, z = 67.9 ± 1.4; left M1: x = −38.3 ± 5.0, y = −19.4 ± 6.1, z = 58.7 ± 3.0; and right dPMc: x = 22.2 ± 6.8, y = −3.5 ± 7.2, z = 63.5 ± 7.4) and then reconstructed on a standard template using MRIcron software (v 1.40 http://www.mricro.com). (**c**) Experiment timeline.

Twelve right-handed healthy participants took part in an experimental session and a control session performed on two separate days, during which MEPs induced by a TS delivered over the left M1 were collected from the right first dorsal interosseous (FDI). In the experimental session, we performed 4 experimental blocks, differing as a function of the CS site (pIFG or pre-SMA) and CS intensity (subthreshold: 90% rMT; or suprathreshold: 110% rMT) (see Fig. 1). In each experimental block, we randomly intermixed spTMS trials (TS alone) and dsTMS trials (TS preceded by a CS with an ISI randomly set at 40, 60, 80, 100, 120 or 150 ms). In this way, we investigated pIFG-M1 and pre-SMA-M1 intensity-dependent causal interactions and identified temporal windows sensitive to the influence of premotor conditioning over M1 excitability. A control experiment was performed by administering the TS over the left M1 and the CS over the contralateral (right) dorsal premotor cortex (dPMc) (Fig. 1; see below for details regarding the control session). Both in the experimental and control sessions, MEPs induced by spTMS were collected in two separate blocks serving as a baseline. In order to normalize the distribution of MEP data collected in each experimental and control block, we computed the mean MEP amplitude value across the two spTMS baseline blocks for each participant (see Methods); then we used this baseline value to express relative changes (% of baseline) in MEP amplitudes induced by each spTMS and dsTMS condition within each experimental and control block.

### Identification of critical ISIs: pIFG-M1 experimental blocks

**Figure 2:**
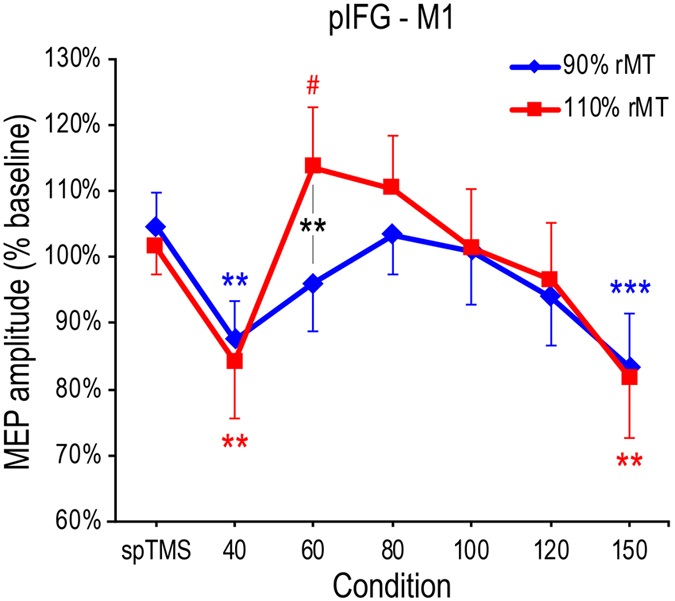
Normalized MEP amplitudes (% baseline) in the pIFG-M1 blocks. The graph illustrates the CS intensity (90% and 110% of rMT) × Condition (spTMS, and dsTMS with 40-150-ms ISIs) interaction. Error bars denote s.e.m. Hash marks and asterisks indicate marginally significant and significant post-hoc comparisons, respectively (Newman-Keuls test, ^#^P < 0.06, *P < 0.05, ***P < 0.001).

To explore intensity-dependent causal interactions from pIFG to M1, dsTMS was performed in two experimental blocks where participants received a TS over the left M1 preceded by a CS over the ipsilateral pIFG either at a subthreshold or a suprathreshold CS intensity. A CS intensity (2 levels: subthreshold and suprathreshold) × Condition (7 levels: spTMS, and dsTMS with 40–150-ms ISIs) ANOVA conducted on normalized MEP amplitudes (% of baseline) showed a main effect of Condition (F_6,66_ = 4.19, P = 0.001; *Partial eta*^2^ = 0.28) while the main effect of CS intensity did not reach significance (F_1,11_ = 0.18, P = 0.68). However, we found a CS intensity × Condition interaction (F_6,66_ = 2.90, P = 0.014; *Partial eta*^2^ = 0.21; Fig. 2), showing that the modulatory effect of dsTMS depended on CS intensity. A post-hoc analysis (performed with Newman-Keuls test) was used to identify critical ISIs at which MEPs evoked by dsTMS differed from MEPs evoked by spTMS, and to check the influence of CS intensity.

In the subthreshold CS intensity block, MEPs in the dsTMS conditions at 40- and 150-ms ISIs (mean amplitude relative to the baseline: 87.5% and 83.4%, respectively) were lower than MEPs in the spTMS condition (104.6%; all P < 0.008). Similarly, in the suprathreshold CS intensity block, MEPs in the dsTMS conditions at 40- and 150-ms ISIs (84.1% and 81.7%, respectively) were lower than MEPs in the spTMS condition (101.5%; all P < 0.005). Moreover, MEP amplitudes induced by dsTMS at these two ISIs were comparable for subthreshold and suprathreshold CS intensity (all P > 0.44). Interestingly, in the suprathreshold CS block, dsTMS MEP amplitudes at an ISI of 60 ms were marginally larger than spTMS MEP amplitudes (113.6% vs. 101.5%; P = 0.057) and significantly larger than dsTMS MEP amplitudes collected in the subthreshold CS block at the same ISI (113.6% vs. 95.9%; P = 0.005), indicating timing-specific dsTMS intensity-dependent effects. No other significant comparisons were found (P > 0.19).

We further explored the dsTMS effects at an ISI of 60 ms by using a more lenient post-hoc test (Duncan test). This showed that, relative to the MEP amplitudes in the spTMS conditions, MEP amplitudes in the dsTMS condition at a 60-ms ISI were significantly larger following a suprathreshold CS (P = 0.015) but tended to be suppressed by a subthreshold CS (P = 0.095). These findings should be interpreted with caution as they show a non-significant trend detected with a less conservative post-hoc test, and future investigations should ascertain the validity of this trend. If confirmed, it would provide further support to the notion that dsTMS exerts timing-specific and intensity-dependent facilitatory and inhibitory effects over the pIFC-M1 circuit driven by supra- and subthreshold CS, respectively.

### Identification of critical ISIs: pre-SMA-M1 experimental blocks

**Figure 3:**
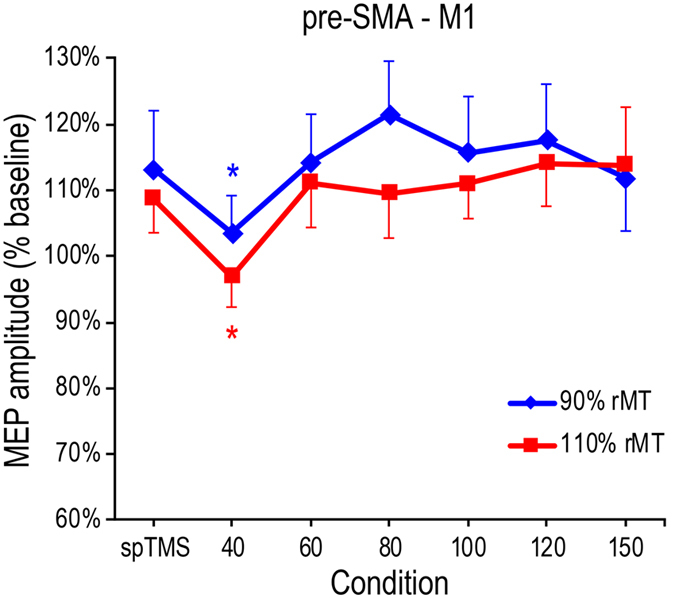
Normalized MEP amplitudes (% baseline) in the pre-SMA-M1 blocks . The graph illustrates a non-significant CS intensity (90% and 110% of rMT) × Condition (spTMS, and dsTMS with 40-150-ms ISIs) interaction. Error bars denote s.e.m. Asterisks indicate significant post-hoc comparisons (Newman-Keuls test, *P < 0.05).

To investigate causal interactions from pre-SMA to M1, participants were also tested in two additional experimental blocks in which subthreshold (90% of rMT) or suprathreshold (110% of rMT) CS intensities were administered over the pre-SMA. The CS intensity (2 levels: subthreshold and suprathreshold) × Condition (7 levels: spTMS, and dsTMS with 40–150-ms ISIs) ANOVA conducted on normalized MEP amplitudes (% of baseline) showed a main effect of Condition (F_6,66_ = 3.02, P = 0.011, *Partial eta*^2^ = 0.22; Fig. 3) accounted for by the significant decrease in MEP amplitudes between the spTMS condition and the dsTMS condition at an ISI of 40 ms (110.9% vs. 100.2%; P = 0.016). No other dsTMS conditions (i.e., ISIs 60–150 ms) were different from the spTMS condition (all P > 0.68). Neither the main effect of CS intensity nor the CS site x CS intensity interaction was significant in the ANOVA (F < 0.73, P > 0.41).

### Control session: dPMc-M1 control blocks

The analysis of MEPs in the experimental blocks revealed that the CS over both the pIFG and the pre-SMA reduced MEP amplitudes at a 40-ms ISI. To rule out that this inhibitory modulation was due to nonspecific effects (e.g., the coil click)^43^,^44^, participants were further tested in a control session on a separate day. This included two short counterbalanced control blocks in which subthreshold (90% of rMT) or suprathreshold (110% of rMT) CS intensities were applied over a brain region that is not believed to influence motor excitability at about a 40 ms ISI (at least when using CS intensities similar to those used here), namely, the contralateral (right) dPMc^28^,^37^. Both dPMc-M1 control blocks included dsTMS trials (a TS preceded by a CS with an ISI of 40 ms) randomly intermixed with spTMS trials (TS alone).

The CS intensity (2 levels: subthreshold and suprathreshold) × Condition (2 levels: spTMS, and dsTMS with a 40 ms ISI) ANOVA conducted on normalized MEP amplitudes (% of baseline) showed no significant effects (F < 0.93, P > 0.36) confirming the lack of dPMc influence over M1 at an ISI of 40 ms.

### Comparing ISI-specific modulatory effects in premotor-motor circuits

The two main analyses detected three critical ISIs at which dsTMS revealed clear modulatory effects of at least one CS site over M1 excitability, i.e., 40, 60 and 150 ms. To directly compare such effects in the two pIFG-M1 and pre-SMA-M1 circuits, for each experimental block and critical ISI, we computed a modulation index on normalized MEPs (% of baseline) as the difference between dsTMS MEPs and spTMS MEPs of the same block. Then we submitted this index to a series of CS site x CS intensity ANOVAs, one for each critical ISI.

**Figure 4:**
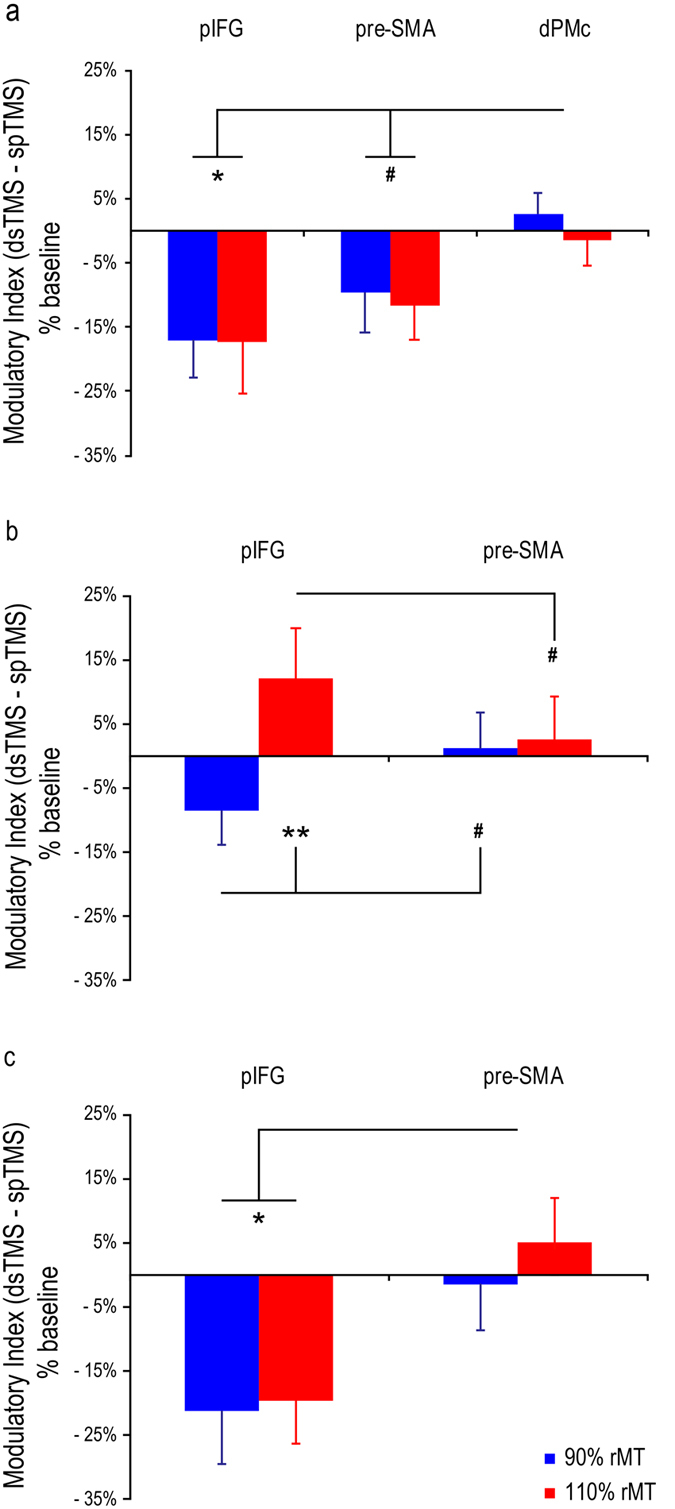
Modulatory effects revealed by dsTMS (dsTMS minus spTMS normalized MEP amplitudes) in the targeted areas at each critical ISI. (**a**) 40-ms ISI, including data from the control experiment; (**b**) 60-ms ISI; (**c**) 150-ms ISI. Error bars denote s.e.m. Hash marks and asterisks indicate marginally significant and significant post-hoc comparisons, respectively (Newman-Keuls test, ^#^P < 0.07, *P < 0.05, **P < 0.01).

In the earliest, 40-ms ISI, the main analyses reported above revealed inhibitory effects in both pIFG-M1 and pre-SMA-M1 circuits. To test site-specificity, we analyzed the modulation index computed at the 40-ms ISI using a CS site (2 levels: pIFG and pre-SMA) × CS intensity (2 levels: subthreshold and suprathreshold) ANOVA. This analysis did not show any main effects or interactions (F < 0.84, P > 0.38), suggesting the inhibitory influence of premotor stimulation at an ISI of 40 ms was comparable across pIFG/pre-SMA sites and sub/suprathreshold CS intensities. Then, we included data from the control experiment in a CS site (3 levels: pIFG, pre-SMA, dPMc) × CS intensity (2 levels: subthreshold and suprathreshold) ANOVA. This second analysis showed the main effect of the CS site (F_2,22_ = 5.15, P = 0.015, *Partial eta*^2^ = 0.32; Fig. 4a), but not the main effect of CS intensity, nor a CS site x CS intensity interaction (F < 0.18, P > 0.67). Post-hoc tests (Newman-Keuls) revealed a significant difference between pIFG and dPMc (mean modulatory indices: −17.2% vs. 0.6%; P = 0.012) and a nearly significant difference between pre-SMA and dPMc (−10.7% vs. 0.6%; P = 0.057), both indicating stronger M1 suppression for pIFG and pre-SMA conditioning than for dPMc conditioning when the critical 40-ms ISI was tested.

At an ISI of 60 ms, the main analysis reported in the previous paragraph revealed an intensity-dependent modulation in the pIFG-M1 circuit but not in the pre-SMA-M1 circuit. To test site-specificity, we performed a CS site (2 levels: pIFG and pre-SMA) × CS intensity (2 levels: subthreshold and suprathreshold) ANOVA on the modulation index. The analysis showed a significant interaction (F_1,11_ = 8.00, P = 0.016, *Partial eta*^2^ = 0.42; Fig. 4b), suggesting a differential impact of CS intensity depending on the CS site. The post-hoc analysis showed that when the CS was administered over the pIFG site, the modulatory index was greater for suprathreshold than for subthreshold CS intensity (12.2% vs. −8.7%; P = 0.006), whereas the modulatory index was comparable with suprathreshold and subthreshold pre-SMA conditioning (2.5% and 1.1%; P = 0.78). Additionally, the modulatory index tended to be larger for pIFG than for pre-SMA conditioning when a suprathreshold intensity was used (P = 0.07), whereas it tended to be lower for pIFG than for pre-SMA when a subthreshold intensity was used (P = 0.07). The two main effects were non-significant (F < 2.10, P > 0.18).

Finally, at a 150-ms ISI, the main analysis showed a reduction in MEPs when subthreshold or suprathreshold CS intensities were administered over pIFG, but not over pre-SMA. The CS site (2 levels: pIFG and pre-SMA) × CS intensity (2 levels: subthreshold and suprathreshold) ANOVA on the modulation index demonstrated site-specific modulation by showing a significant main effect of the CS site (F_1,11_ = 8.80, P = 0.013, *Partial eta*^2^ = 0.44; Fig. 4c). This indicates stronger suppression for pIFG (−20.5%) than for pre-SMA conditioning (1.9%) at a 150-ms ISI. Neither the main effect of CS intensity nor the CS site x CS intensity interaction was significant (F < 1, P > 0.58).

## Discussion

The causal interactions between pIFG and M1 or pre-SMA and M1 are still scarcely known, since the available dsTMS data mostly pertain to short temporal windows (CS-TS ISI < 15 ms) that are supposed to tap into direct anatomical connections. Here we have shown that long-latency pIFG-M1 and pre-SMA-M1 connections also robustly influence M1 output, likely through indirect pathways. We performed a systematic dsTMS investigation of the pIFG-M1 and pre-SMA-M1 circuits, and tested their interactions using a wider temporal window (with ISIs ranging from 40 to 150 ms) and varying CS intensities (90 or 110% of the rMT). Our findings revealed several distinct time intervals at which pIFG and pre-SMA influence M1 output during a resting state. Specifically, three critical time intervals of rostral premotor-motor interactions were revealed, corresponding to ISIs of 40, 60 and 150 ms. These timings showed different site-specific and intensity-dependent effects of the CS on the amplitude of MEPs evoked by left M1 stimulation.

A strong modulatory influence of premotor stimulation over M1 activity was found in the earliest tested time interval (i.e., when the CS was administered over pIFG or pre-SMA 40 ms prior to the TS). To rule out the possibility that these inhibitory modulations were due to nonspecific effects such as the coil click or TMS-related somatosensory stimulation of the scalp, a control experiment targeting the right dPMc was performed. The results showed that dsTMS over dPMc-M1 at a 40-ms ISI did not modulate MEPs, relative to those evoked by spTMS (TS alone). Similar null findings with dsTMS at a 40-ms ISI have been reported in previous studies when the CS was administered to parietal or (pre)motor control areas at CS intensities similar to those used here^45^,^46^. Taken together, the previous and present findings suggest that the MEP reduction found at a 40-ms ISI reflects anatomic- and time-specific rostral premotor-motor connectivity and cannot be attributed to nonspecific effects.

The 40-ms ISI appeared to be a key time-interval for highlighting both pIFG-M1 and pre-SMA-M1 interactions, despite the functional differences shown by these areas within the motor network at different ISIs. It is worth noting that M1 modulation by pre-SMA conditioning occurred only with a 40-ms ISI, while longer ISIs did not significantly affect M1 excitability. A different pattern of modulatory causal influence could be observed following pIFG conditioning at longer ISIs. Indeed, pIFG stimulation brought about a second peak of intensity-independent inhibition when the CS was delivered 150 ms before M1 stimulation. Interestingly, at a 60-ms ISI, we also observed a CS intensity-dependent M1 modulation due to pIFG conditioning. The direction of the modulation that pIFG exerts over M1 was contingent upon the CS intensity applied: M1 excitability tended to be enhanced only if the CS had a suprathreshold intensity. Moreover, using a more lenient post-hoc correction, we found a tendency for suppression with a subthreshold CS at this ISI. Although these trends should be interpreted with caution, intensity-dependent effects at the 60-ms ISI were specific to the pIFC-M1 circuit, as no similar modulations were detected with pre-SMA conditioning.

This pattern of pIFG-M1 interactions fits with the well-known role of the pIFG in regulating motor output. Neurophysiological studies in human and non-human primates suggest that posterior inferior frontal regions are involved in action planning and exert fine-tuned control over M1 by transforming sensory information into specific motor programs^1^,^12^,^47^,^48^. Importantly, these studies indicate that connections between inferior frontal regions and M1 are critically involved in conveying information used to optimally adapt hand configuration to the object to be grasped, providing evidence that these connections play an important role in the fine control of low-level motor parameters^1^,^4^,^12^,^48^. This appears in line with our data showing that the pIFG exerted a time- and intensity-dependent excitatory and inhibitory influence over M1 excitability, and with the fact that this such pIFG influence could be found well before M1 stimulation (i.e., with the 150-ms ISI).

Intensity-dependent bidirectional facilitatory and inhibitory influences have been reported in studies exploring short-latency pIFG-M1 interactions (e.g., at an ISI of 4–6 ms)^33^. The intensity-dependent switch in the net modulatory effect of posterior inferior frontal cortex stimulation has been interpreted as recruitment of different classes of intra-cortical interneurons in M1^33^, possibly due to the activation of different neural populations with different activation thresholds in the pIFG. This explanation is supported by monkey studies showing that, while connections between the premotor cortex and M1 are excitatory, specifically glutamatergic, there are, nonetheless, synapses on both pyramidal neurons and inhibitory interneurons within M1^13^. Thus, the highlighted pattern of CS intensity dependence may reflect distinct involvements of underlying inhibitory and facilitatory pIFG-M1 circuits. They may implicate distinct intra-cortical M1 interneurons, but also third cortical or subcortical structures, considering the long-latency timings explored in the present study. Gerloff *et al*.^49^ suggested that long-latency interhemispheric interactions (with ISIs > 50 ms) might be mediated, to a certain extent, by subcortical regions. In keeping with this idea, Neubert and colleagues^50^, combining dsTMS and diffusion-weighted magnetic resonance imaging, suggested that subcortical pathways involving the basal ganglia mediate interactions between the pIFG and contralateral M1 conducive to action reprogramming at relatively early latencies (ISI of 12 ms). Admittedly, the CS-induced modulations of MEPs at these long latencies might not be solely ascribed to direct connections between the conditioning brain site and M1, but might be based on the recruitment of larger scale CS-related brain networks involving indirect pathways^51^,^52^. Our data do not provide any information about the specific pathway involved in the long-latency influence of pIFG or pre-SMA over M1 and this represents a potential limitation of our study. However, it appears that these routes are at least partially separate, considering the site-specific effects in our results.

Intensity-dependent bidirectional pIFG-M1 influences may reflect mechanisms for action control, as suggested by previous dsTMS studies addressing short-latency (6–8 ms ISIs) pIFG-M1 interactions during active tasks: inhibitory modulations typical of the resting state turn into facilitations during action planning and execution^31^,^53^. Similarly, in action selection, pIFG facilitatory effects turn into inhibitory effects during action reprogramming, when contextual information prompts a switch to a different motor response^50^. Thus, the fine-grained regulation of M1 output, as a consequence of the CS intensity used over pIFG, supports the notion that the pIFG acts as a modulator, able to activate different cells and generate relevant information for M1 to emit a specific motor command.

The pre-SMA stimulation revealed an inhibitory (but not excitatory) influence over M1 only at a 40-ms ISI, regardless of CS intensity, whereas pIFG stimulation showed more complex facilitatory and inhibitory modulations at different time points. This is in keeping with the stronger modulatory effects reported with pIFG stimulation relative to pre-SMA stimulation by Picazio and colleagues at short-time latencies^54^ and further supports the key role of the pIFG in the fine tuning of corticomotor output.

The distinct long-latency influences of the pIFG and the pre-SMA on M1 excitability may reflect their distinct roles in the hierarchy of action control. The frontal lobe is structured as a hierarchy of processes mediating the temporal arrangement and cognitive control of behavior^55^,^56^. A cascade of control processes mediating sensory, contextual and episodic control are implemented in prefrontal and premotor areas. Considering the roles of the pIFG and the pre-SMA in planning and controlling actions^1^,^2^,^3^,^4^,^5^,^6^, it might be suggested that these regions play partially distinct roles in the frontal hierarchy and in the regulation of M1 neurons. While the pIFG is also engaged in relatively simple motor tasks and exerts a fine-tuned modulatory influence over M1 neurons, the pre-SMA is involved in higher-level action planning and plays a particularly prominent role in cognitively demanding motor tasks^2^,^4^,^5^,^57^,^58^. The pre-SMA (and the supplementary motor complex in general) releases high-level commands for subsequent downstream motor processes, and it is supposed to exert an influence over M1 for action initiation. This may explain why the dsTMS protocol in our resting conditions with no active motor task revealed only an influence of the pre-SMA over M1 at the shortest 40-ms ISI which did not depend on CS intensity. However, it could be speculated that earlier (i.e., longer-latency ISIs) and more fine-tuned modulatory influences of the pre-SMA over M1 could be revealed during complex motor tasks, in keeping with a higher-level role for this region in action control. Further studies are needed to test this hypothesis.

In sum, using dsTMS, we revealed the existence of long-latency premotor-motor interactions consisting of modulation of M1 motor output by pIFG or pre-SMA conditioning at critical time intervals. The reported modulations highlight the distinct roles of the pIFG and pre-SMA in causally influencing motor output in resting-state conditions. Moreover, they are consistent with the general concept that investigations of motor connectivity during a resting state can provide insights into the functions of motor networks^18^. Our results show fine-grained premotor modulation of M1 excitability that is site-specific and both time- and intensity-dependent. Investigations of long-latency premotor-M1 interactions are important for understanding cortico-cortical connectivity at rest, and can pave the way for future investigations during active motor tasks and/or cognitive tasks where premotor-motor connectivity might be involved^30^,^59^,^60^,^61^. Moreover, tracking the specific time courses of pIFG-M1 and pre-SMA-M1 interactions in the healthy brain can pave the way for investigations of pathological conditions. While our study does not provide evidence for the specific pathways that might mediate these neurophysiological interactions, our data allow us to identify specific time intervals in which premotor regions can influence M1 output. These time intervals are of potential interest, as they may be amenable to connectivity manipulations, for example, via the cc-PAS protocol, which relies on the critical ISIs identified by dsTMS data^39^,^40^,^41^,^42^. Future applications of these protocols may be promising for clinical conditions where connectivity across functional networks is altered^18^,^62^,^63^,^64^.

## Materials and Methods

### Participants

Twelve healthy volunteers (7 females; mean age ± S.D.; 24.8 ± 2 years), free of any contraindications to TMS^65^ gave written informed consent prior to the study. All participants were right-handed according to the Edinburgh Handedness Inventory^66^. The experimental protocol was approved by the Bioethics committee of the University of Bologna and was carried out in agreement with legal requirements and international norms (Declaration of Helsinki, 1964). The methods carried out in this work are in accordance with approved guidelines. None of the participants reported adverse reactions to TMS.

### Experimental procedure

Participants took part in an experimental session and a control session separated by 7 ± 3 days. In both sessions, MEPs evoked by spTMS and dsTMS were recorded. During spTMS trials, a TS pulse was administered alone over the left M1. During dsTMS trials, a TS pulse was administered over the left M1 and preceded by a CS over a target area. Participants sat with both hands relaxed and were instructed to keep their eyes closed with the purpose of obtaining a signal as stable as possible and minimizing the influence of potentially distracting visual stimuli. Electromyographic (EMG) recording was performed through Ag/AgCl surface electrodes placed over the right FDI in a belly-tendon montage. EMG signals were acquired by means of a Biopac MP-35, band-pass filtered (30–500 Hz) and sampled at 5 kHz. TMS pulses were delivered via 2 figure-of-eight coils (50 mm wing coil outer diameter), each of which was connected to a Magstim 200 monophasic stimulator. The left M1 was identified as the hotspot where the TS induced the largest MEP amplitudes with the coil held tangentially to the scalp, at a ~45° angle to the midline, inducing a posterior-to-anterior current^67^,^68^. The TS intensity was set to produce a MEP amplitude of about 1.0–1.5 mV (mean ± S.D.: 51% ± 11 of the maximum stimulator output, MSO).

The experimental session consisted of 4 experimental blocks testing pIFG-M1 interactions (in two blocks) or pre-SMA interactions (in the other two blocks). The control session consisted of 2 control blocks testing dPMc-M1 interactions. For each stimulated area, 2 CS intensities were used (i.e., 90% or 110% of rMT) and were tested in separate blocks. The rMT was defined as the minimum stimulator output intensity that induced a MEP with >50 μV amplitude in 5 out of 10 consecutive trials^68^. The mean rMT was 40% ± 7 of the maximum stimulator output. Each of the experimental blocks included 152 trials (32 spTMS trials and 120 dsTMS trials: 20 trials for each of the 6 ISIs, i.e., 40, 60, 80, 100, 120 and 150 ms). Each of the 2 control blocks included 52 trials (32 trials of spTMS and 20 trials of dsTMS using a 40-ms ISI). Block and trial orders were randomized. Additionally, at the beginning of the first session (either the experimental or the control session) we collected a block of 10 spTMS trials constituting the baseline/pre block; at the end of the second session, we collected another block of 10 spTMS trials, constituting the baseline/post block.

The control session was motivated by a preliminary off-line analysis performed on data from 7 participants who were initially tested in the experimental session only. This analysis revealed that the CS over both the pIFG and the pre-SMA tended to consistently reduce MEPs at a 40-ms ISI. Thus, to rule out that this inhibitory modulation was due to nonspecific effects (e.g., the coil click), we tested these participants in the control session, in which a CS was applied over the dPMc. These seven participants were tested first in the main experiment and then in the control experiment. The remaining participants were tested in the opposite order.

### Brain localization

Brain conditioning sites were identified using established methods. The left pIFG location was identified with the EMS SofTaxic Navigator system, which automatically estimates coordinates in Talairach space from a magnetic resonance imaging-constructed stereotaxic template. Skull landmarks and ~80 points providing a uniform representation of the scalp were digitized by means of a Northern Digital Polaris Vicra digitizer^69^,^70^,^71^. An individual estimated magnetic resonance image (MRI) was obtained for each subject through a 3D warping procedure fitting a high-resolution MRI template with the participant’s scalp model and craniometric points. This procedure ensures a global localization accuracy of ~5 mm^69^. We targeted the pIFG using the following Talairach coordinates: x = −54, y = 10, z = 24^72^,^73^. The coil was placed at ~45° to the midline to induce a ventro-lateral to medio-posterior current^33^. Based on previous research, we used craniometric methods to identify the pre-SMA and dPMc scalp positions. The pre-SMA was stimulated 4 cm anterior to the vertex on the sagittal midline as in previous research^15^,^35^,^40^, with the coil handle pointing forward to induce an anterior-posterior current^35^. The right dPMc was stimulated 2 cm anterior and 1 cm medial with respect to the right M1 hotspot for inducing MEPs in the left FDI, and the coil was held at ~90° from the midline, inducing a latero-medial current^29^,^36^,^74^.

The SofTaxic Navigator system was used to estimate the projection of the targeted scalp positions on the brain surface, confirming correct coil placement for all the sites^69^,^70^,^71^,^72^,^73^. The estimated Talairach coordinates for the left M1 (i.e., the FDI optimal scalp position) were (mean ± S.D.): x = −38.3 ± 5.0, y = −19.4 ± 6.1, z = 58.7 ± 3.0. Brain surface Talairach coordinates for the pIFG were: x = −54.8 ± 1.3, y = 9.1 ± 1.0, z = 24 ± 1.0; coordinates for the pre-SMA were: x = 0.1 ± 0.3, y = 9.8 ± 6.5, z = 67.9 ± 1.4; right dPMc: x = 22.2 ± 6.8, y = −3.5 ± 7.2, z = 63.5 ± 7.4 (See Fig. 1).

### Data analysis

In each block, the mean peak-to-peak MEP amplitude was computed for the spTMS condition and each dsTMS condition. Any trace showing EMG activity 100 ms prior to the TMS pulses was excluded (~4%). In each condition, MEPs with amplitudes deviating from the mean by more than 2.5 S.D. were removed from the analysis (~3%). A preliminary one-way ANOVA was conducted on mean MEPs elicited by spTMS in all the experimental, control and baseline blocks (8 levels: pIFG/subthreshold, pIFG/suprathreshold, pre-SMA/subthreshold, pre-SMA/suprathreshold, dPMc/subthreshold, dPMc/suprathreshold, baseline/pre, baseline/post). The ANOVA was not significant (F_7,88_ = 1.01, P = 0.43), indicating that motor excitability measured by spTMS stimulation was comparable across experimental, control and baseline blocks. For each participant, we averaged MEPs across the pre- and post-baseline blocks and used this value to normalize MEP amplitudes in the different conditions of each experimental block (i.e., spTMS-MEPs, and dsTMS-MEPs at ISIs from 40 to 150 ms were divided by the baseline spTMS-MEPs) and control block (i.e., spTMS-MEPs and dsTMS-MEPs at a 40-ms ISI were divided by the baseline spTMS-MEPs).

Two separate CS intensity (subthreshold and suprathreshold) × Condition (spTMS, and dsTMS at ISIs from 40 to 150 ms) ANOVAs were performed on normalized MEP amplitudes (% of baseline), one for each conditioned area (pIFG and pre-SMA). A post-hoc analysis was performed with the Newman-Keuls test in order to compare dsTMS-MEPs relative to spTMS-MEPs within each area, and to correct for multiple comparisons. This analysis revealed the critical ISIs at which a CS over a target region influenced M1 excitability. To compare the modulatory effects revealed by dsTMS in the different areas, we also subtracted normalized MEP amplitudes in the spTMS condition from those in the dsTMS condition, in order to directly compare inhibitory/facilitatory effects in the pIFG-M1 and pre-SMA-M1 circuits. Subsequently we submitted these modulation indices to a series of Area x CS intensity ANOVAs, one for each critical ISI.

Data from the control experiment were analyzed following the same procedure used for data from the experimental session. Thus, MEPs elicited by spTMS and dsTMS were normalized using the previously computed grand average baseline, and submitted to a CS intensity (subthreshold and suprathreshold) × Condition (spTMS, and dsTMS at a 40-ms ISI) ANOVA. Moreover, to compare the modulatory effect (dsTMS minus spTMS normalized MEP amplitudes) induced by dsTMS stimulation at a 40-ms ISI with the brain stimulation sites examined in the experimental session, a further Area (pIFG, pre-SMA, dPMc) × CS intensity (subthreshold and suprathreshold) ANOVA was computed.

## Additional Information

**How to cite this article**: Fiori, F. *et al*. Long-latency modulation of motor cortex excitability by ipsilateral posterior inferior frontal gyrus and pre-supplementary motor area. *Sci. Rep.* **6**, 38396; doi: 10.1038/srep38396 (2016).

**Publisher's note:** Springer Nature remains neutral with regard to jurisdictional claims in published maps and institutional affiliations.
